# Bacterial Behavior in Confined Spaces

**DOI:** 10.3389/fcell.2021.629820

**Published:** 2021-03-18

**Authors:** Hang Du, Weili Xu, Zhizhou Zhang, Xiaojun Han

**Affiliations:** ^1^State Key Laboratory of Urban Water Resource and Environment, School of Chemistry and Chemical Engineering, Harbin Institute of Technology, Harbin, China; ^2^Center for Marine Antifouling Engineering Technology of Shandong Province, School of Marine Science and Technology, Harbin Institute of Technology, Weihai, China

**Keywords:** bacterial behavior, confined space, growth and proliferation, communication, motion

## Abstract

In confined spaces, bacteria exhibit unexpected cellular behaviors that are related to the biogeochemical cycle and human health. Types of confined spaces include lipid vesicles, polymer vesicles, emulsion droplets, microfluidic chips, and various laboratory-made chambers. This mini-review summarizes the behaviors of living bacteria in these confined spaces, including (a) growth and proliferation, (b) cell communication, and (c) motion. Future trends and challenges are also discussed in this paper.

## Introduction

The human body is a representative host of microorganisms. A wide range of bacteria exist in the human intestine and oral cavity and are closely related to human health. The functional behaviors of these bacteria, including oil extraction, nitrogen fixation, and bioremediation, all take place in narrow spaces. Therefore, the study of bacteria in confined spaces is of great significance with respect to the biogeochemical cycle and the healthy balance of the human microbial community. Currently, there is very limited knowledge regarding living matter in confined spaces. Observations and multi-disciplinary investigations of bacterial behaviors in confined spaces have attracted intense interest in recent years.

In the natural environment, the same bacteria may show completely different behaviors in different habitats. The relevant types of behaviors are (a) growth and proliferation, (b) communication, and (c) motion. The habitats are often confined spaces, such as injury sites or intestines. Investigation of bacterial behaviors in restricted spaces will help us to more accurately analyze the factors influencing these behaviors. A confined space usually has good packaging and physical isolation, preventing external interference in bacterial behaviors. Such confined spaces include phospholipid vesicles (Elani et al., 2018; Trantidou et al., 2018), polymer vesicles (Meyer et al., 2015; Luo et al., 2020), emulsion droplets (Zhang et al., 2013; Mahler et al., 2018), and microfluidic chips (Inoue et al., 2001; Keymer et al., 2006).

Herein, we summarize recent research progress on three types of bacteria behaviors in confined spaces: growth and proliferation, communication, and motion. Future prospects are discussed at the end of the paper.

## Confined Spaces

Bacteria are approximately several microns in size. To study the behavior of bacteria more precisely, confined spaces can be designed to range from several microns to tens of microns. These confined spaces can be roughly divided into four categories: phospholipid vesicles, polymer vesicles, droplets, and microfluidic chips. Phospholipid vesicles can be fabricated by hydration and phase transfer methods, and they serve as a culture environment for bacterial cells. Methods to prepare polymer vesicles largely depend on the vesicles’ compositions. The main polymers used to encapsulate bacteria are gels and porous materials. Droplets can be obtained by mixing the bacterial solution with oils or using microfluidic techniques. Microfluidic chips are fabricated by typical photolithography methods. Table 1 summarizes studies of bacterial behavior in the abovementioned confined spaces.

**TABLE 1:** Types of bacterial behaviors inside confined spaces.

Confined space	Bacterium	Types of bacterial behavior	Applications	Unexpected behavior (in contrast with traditional methods)	Example references
Lipid vesicles	*E. coli*	(a) Growth and proliferation	To observe bacterial growth and proliferation	The bacteria proliferated more slowly.	Morita et al., 2018
	*E. coli*	(a) Growth and proliferation	To monitor microbial growth and proliferation	*E. coli* proliferated more slowly, whereas other bacteria elongated without division owing to the accumulation of matter.	Juskova et al., 2019
Polymer vesicles	*E. coli*	(a) Growth and proliferation; (b) communication	To investigate effects of physical barriers against mass gain and cell division	Both the GFP fluorescent signal of bacteria and the cell size increased by factors of more than five and two in the confined space, respectively.	Rybkin et al., 2019
Droplets	*Staphylococcus aureus*	(b) Communication	To study confinement-induced QS	Individual or small groups of *S. aureus* bacteria initiated virulence factor expression.	Carnes et al., 2010
	*E. coli* and *B. subtilis*	(c) Motion	To observe the swimming behavior of bacteria	Dense suspensions of *E. coli* produced notable periodic vortex reversal.	Hamby et al., 2018
	*E. coli*	(c) Motion	To observe the motion of bacteria to transfer mechanical energy to the confining environment	The motion of dense *E. coli* inside the droplet propelled droplet movement.	Ramos et al., 2020
	*Magnetospirillum gryphiswaldense*	(c) Motion	To observe the bacteria self-assembling into a rotating body in a confined space	Living bacteria self-assembled into a rotary motor.	Vincenti et al., 2019
	Two *B. subtilis* strains: the WT 168 and the mutant strain	(c) Motion	To measure the swimming and motion directions of bacterial cells	The microorganisms swam upstream against the spiral vortex.	Lushi et al., 2014
	*Pseudomonas aeruginosa*	(b) Communication	To study the QS pathways of bacteria	One to three bacteria initiated QS and achieved QS-dependent growth.	Boedicker et al., 2009
Microfluidic chips	*Salmonella typhimurium*	(b) Communication	To observe bacterial cancer-targeting to normal (THLE-2) or cancer hepatocytes (HepG2)	*S. typhimurium* showed a significant preference for HepG2 cells compared with normal hepatocytes.	Hong et al., 2013
	*E. coli*	(b) Communication	To study the adaptive dynamics of the bacterial metapopulation in heterogeneous habitats	Local bacterial populations coexisted and were weakly coupled with neighbor populations.	Keymer et al., 2006
Porous media	*E. coli*	(c) Motion	To track bacterial motion	Bacteria showed intermittent movement in porous media.	Bhattacharjee and Datta, 2019
	*E. coli* and *Staphylococcus sciuri*	(c) Motion	To observe the effects of bacteria with different motility on vesicle trapping	The bacteria with higher activity more easily passed through pores.	Luo et al., 2020

## Bacterial Growth and Proliferation in Confined Spaces

Growth and proliferation are basic behaviors of bacteria. These behaviors in a confined space are different from those observed conventionally.

**FIGURE 1:**
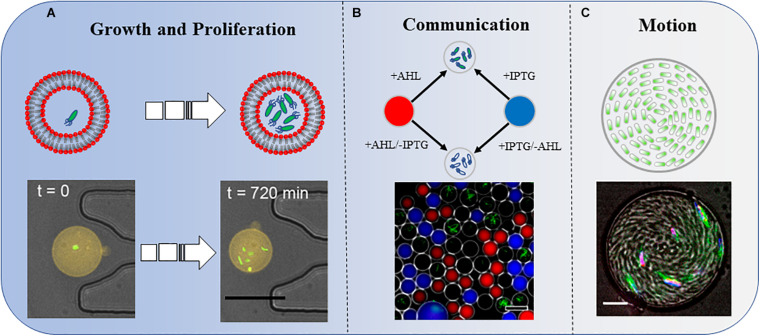
Three types of bacterial behaviors in a confined space. **(A)** Growth and proliferation. Bacterial growth and proliferation in phospholipid vesicles. The shrinkage of the space reduces the growth and proliferation speed of bacteria compared with those in the same culture medium in a non-enclosed space. Scale bar, 25 μm. Adapted with permission from Juskova et al. (2019). Copyright 2019, American Chemical Society. **(B)** Communication. Bacteria sense IPTG and QS molecule AHL diffused into the droplets. The combined effects of these two molecules induce the bacteria to produce GFP. Scale bar, 50 μm. Adapted with permission from Weitz et al. (2014). Copyright 2014, American Chemical Society. **(C)** Motion. A concentrated suspension of bacteria forms a vortex motion in the droplet. Scale bar, 10 μm. Adapted with permission from Lushi et al. (2014). Copyright 2014, National Academy of Sciences.

Juskova et al. (2019) provided a novel method to monitor the real-time activity of bacterial growth inside giant unilamellar vesicles (GUVs) (Figure 1A). They reported the encapsulation of single bacteria in small-volume GUVs (1–33 pL), followed by immobilization of the GUVs on a planar lipid bilayer membrane on a glass surface, under which photoelectronic detection could be applied at a single-cell level (Morita et al., 2018). Polyelectrolyte capsules with different numbers of layers have also been used to encapsulate bacteria. The delayed growth of bacteria was significantly correlated with the number of layers of the reticular vesicles. Polyethylene capsules with more than six layers were very solid. Interestingly, cell size and green fluorescent protein (GFP) expression were increased in thicker polyethylene capsules. This was the first demonstration that confined spaces may affect biochemical reactions in bacteria (Rybkin et al., 2019). In microfluidic channels, owing to continuous cell propagation, *Escherichia coli* is greatly compressed in submicron narrow channels that are smaller than its diameter. Although *E. coli* can still grow and divide, this deformation is irreversible, even if the bacteria are later removed from the confined space (Jakiela et al., 2013).

Some other interesting bacterial behaviors may be observed in confined spaces. For example, when riboflavin-producing bacteria are encapsulated, riboflavin accumulates in vesicles, causing the bacteria to elongate without division (Juskova et al., 2019). Fungi in microfluidic chips have different growth behaviors owing to the limited space. Three conditions were used to explore the mechanism of hyphal extension in a confined space. When fungal hyphae met sharp-angle obstacles, they tended to change direction to avoid the obstacles. When they collided with an obstacle nearly orthogonally, they continued to extend forward after splitting into two hyphae. Finally, the hyphae did not grow after touching the wall (Held et al., 2019).

Studies of bacterial growth and proliferation in confined spaces are at a preliminary stage, as only a few bacterial species have been tested (Table 1). Reports on human cancer cells in confined spaces are still rare (Hong et al., 2013). It is difficult for traditional (low-resolution) methods to simultaneously monitor the biological processes of many cells at single-cell resolution. X-ray crystallography and electron microscopy have for many years been used to capture information on the nanometer scale; however, they are only suitable for static observation. In 2020, a resolution of 1.25 Å was achieved with cryo-electron microscopy (Nakane et al., 2020), which is good enough to pinpoint the position of a single atom in a protein. However, X-ray crystallography and electron microscopy do not work in a high-throughput manner and cannot be used to directly observe living cells. Instead, high-throughput cell studies can be carried out in the confined spaces of a microfluidic chip; this may play an indispensable part by generating a huge amount of new data and numerous novel findings.

## Bacterial Communication in Confined Spaces

In nature, bacteria communicate through the perception and secretion of metabolic signals. Some special bacteria have the ability to communicate through magnetic fields (Shcheglov et al., 2002; Vinhas et al., 2020). There are many ways for bacteria to interact, including chemotactic swimming through signaling molecules and quorum-sensing (QS) molecules, and co-cultivation of two defective bacteria that benefit from their respective metabolites. In the past few years, these methods have been used to observe molecular communications among bacteria in different confined chambers.

Quorum-sensing is a phenomenon that occurs among microorganisms with a high population density. The signaling molecules released by bacteria themselves reach a threshold, which in turn affects the expression of specific bacterial genes and regulates the behavior of microorganisms, such as the formation of biofilms, a coordinated response to toxins, and emission of fluorescence. One such signaling molecule is self-inducer AI (auto-inducer, AI). AI can be roughly divided into three categories: (i) high acylserine lactone (AHL) from Gram-negative bacteria; (ii) oligopeptide molecules from Gram-positive bacteria; and (iii) AI-2 from both types of bacteria. Encapsulation of a single or a small number of bacteria in a confined space has been proven to initiate the QS pathway. Bacteria do not need to grow to achieve the high density required to initiate the density-dependent QS reaction (Boedicker et al., 2009).

A single *Staphylococcus aureus* bacterium can detect its restriction after being captured in a confined space and employ QS to activate virulence and metabolic pathways that maintain survival (Carnes et al., 2010). In one study, by analyzing the strength of the fluorescent signal in the droplets, the authors not only investigated the influence of distances on the strength of signal transmission but also added another factor, IPTG, to explore the results of group sensing in space and time (Figure 1B; Weitz et al., 2014).

An L-lysine-deficient variant relies on L-lysine-producing *Corynebacterium glutamicum* to produce glutamate to be cultured on a microfluidic chip (Burmeister et al., 2018). The yeast community and methanogenic community cooperate through metabolite exchanges. This “strong partner mix” can be extended to multiple microorganisms (Momeni et al., 2013). Three microorganisms can also survive in the same microhabitat through mutual support under conditions of limited nutrition. There is no evidence for interaction among nitrogenase, *Bacillus licheniformis*, and Bacteroides communities in nature. However, they can coexist by providing nutrients to each other in separated chambers of microfluidic chips, even if the nutrients in the environment are limited. It is necessary to study how bacterial communities interact and perform community functions in natural ecosystems, and how to maintain species diversity of microbial communities in a confined space (Kim et al., 2008).

Bacterial communication is one of the biological communication types that has been studied in confined spaces. The next step would be tissue communication, which involves many different cells in the same tissue. Partial tissue samples could be assembled with single cells in a confined space, and it would even be possible to synthesize human organs by assembling cells in confined spaces for drug screening. To this end, cellular communication and regulation must be elucidated in much more detail. Molecular communications among metabolic modules and signaling transduction pathways also require further investigation in more delicate microfluidic devices. Confined-space-based studies are suitable for simultaneously observing and analyzing communications among many cancerous and normal cells at the single-cell level.

## Motion of Bacteria in Confined Spaces

The motion of a single bacterium or cell in a tiny tunnel-like confined space is a physiologically significant behavior. For example, a single-celled organism (amoeba) can travel through the olfactory nerve into the frontal lobe of the brain, which represents a typical case of cell motion in a confined space.

The first direct visualization of bacterial motion at single-cell resolution in three-dimensional (3D) porous media was provided by Bhattacharjee and Datta (2019). They found that bacteria did not simply exhibit run-and-tumble motility as commonly assumed but followed a hop-and-trap model. Although interesting, this study did not consider different bacteria types and pore sizes. The microfluidic environment can enhance the diffusion of swimming bacteria. Bacteria in a geometric maze chip showed typical running and rolling motion. However, the bacteria moving in the maze chip showed stronger motility than free bacteria. It is of great medical significance to study the motion of bacteria in a narrow passage in microfluidic chips, for instance, to better understand the spread of bacteria in the process of infection (Weber et al., 2019).

Bacteria explore their natural environment through frequent motion. Frangipane et al. (2019) built a 3D micro-chamber to study the properties of bacterial random-walk paths and reported some novel results. The mean residence time of swimming bacteria inside the artificial complex microstructures was constrained by the sole free surface to perimeter ratio. As a counterintuitive result, bacteria escape faster from chamber structures with a higher density of obstacles, owing to a reduced accessible surface (Frangipane et al., 2019). In another report, *Bacillus subtilis* within a disk-shaped droplet formed a stable vortex that counterrotated at the periphery. However, under similar confinement, *E. coli* displayed a single periodically reversed-direction vortex on a time scale of seconds (Hamby et al., 2018). More interestingly, once the radius of the confinement chamber was below a critical value, *B. subtilis* formed a steady single vortex within a thin cylindrical chamber. The cells within the spiral vortex swam upstream against the counter-rotating flows (Figure 1C; Lushi et al., 2014). Vincenti et al. (2019) reported that motile magnetotactic bacteria confined in water-in-oil droplets self-assembled into a rotary motor under a constant magnetic field.

Chemotaxis is a sensing mechanism by which individual bacterial cells disperse for exogenous sources of nutrients. Researchers found that in the presence of appropriate environmental topologies in a confined space, stressed bacteria formed solitary “moving waves” and that these waves nucleated population collapse of the bacteria into small confining structures, representing a previously unappreciated role of the bacterial chemotaxis system (Park et al., 2003).

Bacteria in some confined spaces not only have their own motion behavior but also promote the motion of the confined space. A dense bacterial suspension of moving bacteria enclosed in droplets drives the droplets with a continuous Brownian motion (Ramos et al., 2020).

Studies of confined-space-based bacterial motion have many applications in biomedical research and significant potential implications. For instance, they provide micron-scale information on processes including the translocation pathogenic microbes in human tissues, life-cycle trafficking of a virus particle in different cells, metastasis of cancerous cells into healthy tissues. There is strong evidence that amyloid plaques in the human brain wrap microbes (Kumar et al., 2016). However, how these microbes (virus, bacterium, or fungus) move through tissues and translocate into the brain remains obscure.

## Other Observed Bacterial Behaviors in Confined Space

Multi-cell interaction is a sub-field of systems biology that has not seen any major breakthrough until now. It is still difficult to describe in detail multi-cell or multi-species interactions in any known natural microbial community. Confined spaces offer a good opportunity to investigate multi-cell interactions. The observed phenomena are not limited to bacterial growth, proliferation, communication, and motion. Preliminary studies have shown that the mutant type of *E. coli* is more dominant than the wild type in a confined space, in contrast to observations in plate colonies (Hol et al., 2015). When nutrients are scarce, they do not compete in the entire microfluidic chip but coexist in the same habitat and occupy different niches (Lambert et al., 2011). Park et al. (2011) demonstrated that droplets could be effectively utilized to co-cultivate two to three different microbes and detect symbiotic relationships. In another example, a lysine-producing *C. glutamicum* strain and a lysine auxotrophic variant of the same species produced a symbiotic interaction, relying on each other’s metabolites (Burmeister et al., 2018). The authors also investigated bacterial conjugation between *E. coli* S17-1 and *Pseudomonas putida* KT2440 cells and showed that direct cell contact was essential for successful gene transfer *via* conjugation. The spatiotemporal dynamics of synthetic microbial consortia in microfluidic devices (Alnahhas et al., 2019) were obtained by co-culturing two different strains of *E. coli* in microfluidic devices. The size of the cell-trapping region was found to be a critical determinant of the spatiotemporal dynamics.

## Discussion

Studies of bacterial behavior within confined spaces provide novel knowledge in the fields of microbiology and medical science. Feasible types of confined macro-scale apparatus include phospholipid vesicles, droplets, polymer vesicles, microfluidic chips, and other chambers. Microfluidic-based devices will probably be the main platform used to provide confined spaces with unlimited structural complexity in the future. In these confined spaces, growth, proliferation, communication, colonization, dispersion, motion, and other bacterial behaviors can be observed, quantified, and modeled in convenient ways. Owing to their delicate inner structure and physical or chemical properties, such confined spaces have a very important role in quantitative studies of the behavior of potentially all types of bacteria. Studies of the behavior of bacteria in confined spaces will undoubtedly be a future trend, with striking results.

## Author Contributions

HD read all the references of this article and wrote the article. WX provided some suggestions for the article. XH and ZZ helped to establish the logical framework and language polishing of the entire article. All authors contributed to the article and approved the submitted version.

## Conflict of Interest

The authors declare that the research was conducted in the absence of any commercial or financial relationships that could be construed as a potential conflict of interest.
